# Incidental retrieval of prior emotion mimicry

**DOI:** 10.1007/s00221-017-4882-y

**Published:** 2017-02-10

**Authors:** Ralph Pawling, Alexander J. Kirkham, Amy E. Hayes, Steven P. Tipper

**Affiliations:** 1 School of Natural Sciences and Psychology, Liverpool John Moores University, Byrom Street, Liverpool, UK; 2 Department of Psychology, University of York, York, UK; 3 School of Sport, Health and Exercise Sciences, Bangor University, Bangor, UK

**Keywords:** Embodied cognition, Memory, Emotion, Psychophysiology

## Abstract

When observing emotional expressions, similar sensorimotor states are activated in the observer, often resulting in physical mimicry. For example, when observing a smile, the zygomaticus muscles associated with smiling are activated in the observer, and when observing a frown, the corrugator brow muscles. We show that the consistency of an individual’s facial emotion, whether they always frown or smile, can be encoded into memory. When the individuals are viewed at a later time expressing no emotion, muscle mimicry of the prior state can be detected, even when the emotion itself is task irrelevant. The results support simulation accounts of memory, where prior embodiments of other’s states during encoding are reactivated when re-encountering a person.

## Introduction

As social animals humans must understand the current states of other’s, predict future actions and retrieve from memory information about a person that might facilitate later interactions. Evidence supports the notion that such abilities may be underpinned by processes of sensorimotor simulation. That is, representing the observed actions (Avenanti et al. 2013) and internal states (Keysers et al. 2010) of others, involves the activation of similar visuomotor and somatosensory states in the observer.

Research into sensorimotor simulation has focused on perception of motor actions, after the discovery of cells in area F5 of the monkey, referred to as ‘mirror neurons’ (Rizzolatti et al. 2001). These cells are active when the animal performs an action, but also when the animal observes the same or similar actions performed by another actor (di Pellegrino et al.^1992^).

Research studies in humans, utilising behavioral (e.g., Bach and Tipper 2007; Griffiths and Tipper 2009), neuromodulatory (e.g., Fadiga et al. 1995), and functional neuroimaging methods (e.g., Oosterhof et al. 2010), provide evidence that observing actions indeed activates similar motor states in the brain of the observer. Similar results have been shown for observation of sensory experiences, such as viewing others receive noxious (Morrison et al. 2004) and non-noxious touch (Keysers et al. 2004; Morrisson et al. 2011).

Recently, interest in the role of simulation in understanding people’s emotions has increased. Facial expressions are a key observable aspect of emotion (Panksepp 2005), and motor simulation may be a route by which an observer understands the meaning of these actions (Niedenthal et al. 2010; Wood et al. 2016). Viewing facial expressions causes automatic imitative responses in the facial muscles of the observer. For example, viewing another person smile causes an almost immediate increase in activation in the zygomaticus muscle of the cheek, whilst viewing a frown activates the corrugator muscle that draws down the brow (e.g., Dimberg et al. 2000, 2002). These changes in activity from the prior state of the musculature are typically very small, non-visible, and only detectable through measurement of the electrical activity at the muscle site, recorded via facial electromyography (EMG) (for discussion of micro-expressions, see Cacioppo et al. 1992; Tassinary and Cacioppo 1992). This ‘facial mimicry’ occurs rapidly, within hundreds of milliseconds (Dimberg 1997). It also appears automatic, occurring even when participants are instructed not to move their faces (Dimberg et al. 2002), or when the emotion viewed is irrelevant to the task in hand (Cannon et al. 2009).

Recent models purport that facial mimicry represents a ‘spill over’ of sensorimotor simulation (Wood et al. 2016). Vicarious activity in sensorimotor regions elicited when viewing a facial expression (Sato et al. 2015; Schilbach et al. 2008) is thought to trigger activation of other brain and body states associated with the emotion being perceived, and this simulation aids the recognition of the emotion. Whilst simulation may not always involve mimicry (Wood et al. 2016), there is evidence that mimicry can augment or empower simulation of other’s emotions (Lee et al. 2013). Blocking mimicry reduces accuracy in decoding the meaning of facial expressions (Ipser and Cook 2016; Maringer et al. 2011; Ponari et al. 2012; Stel and Knippenberg 2008), whilst amplifying mimicry can improve recognition. Participants whose brow muscles had been paralysed via Botox injections performed worse than controls in an emotion recognition task, whilst participants whose facial feedback was amplified with proprioceptive taping performed better (Neal and Chartrand 2011). It is believed that during facial mimicry, afferent feedback from the facial muscles (Price and Harmon-Jones 2015) adds to the overall simulation of the other’s emotion already occurring in sensorimotor regions of the brain (Niedenthal et al. 2010; Wood et al. 2016).

One issue yet to be addressed is whether such simulations might be reinstated as part of long-term memory for other’s emotions. Reactivation accounts of episodic memory suggest that retrieving a memory involves partial reactivation of the brain states active during encoding, including those involved in sensory and motor processing (Barsalou 1999; Buckner and Wheeler 2001; Glenberg 1997, for reviews, see; Buchanan 2007; Danker and Anderson 2010). Evidence for this comes from neuroimaging research showing that retrieval of stimuli previously associated with differential sensory inputs reactivated sensory-specific cortical regions. For example, recalling an object encoded in the form of a picture, as opposed to in word form, reactivated visual cortex (Vaidya et al. 2002); remembering the sound associated with a particular label reactivates areas of auditory cortex (Wheeler et al. 2000); and remembering whether an object was previously presented in the presence of an odor reactivates olfactory cortex (Gottfried et al. 2004; for limitations see; Danker and Anderson 2010, p 6). Similarly, the retrieval from memory of action words activated motor cortices when the words were encoded alongside performance of the action, but not if the words were encoded without action performance (Nyberg et al. 2001). Interestingly, simply imagining the enactment of the action during encoding activated motor areas, which again became active during retrieval. This latter finding is suggestive of the reactivation of prior simulative processes.

In the same manner, might motor simulations of an individual’s emotional state be reactivated upon retrieval of that individual’s identity? According to models, such as that of Wood et al. (2016), viewing another person’s smile should activate the face regions within the viewer’s sensorimotor cortices, and might result in facial mimicry in the zygomaticus muscle, with resultant afferent feedback, creating a simulation. Upon meeting the same individual again, would this simulation then be reinstated as part of implicit recall of the person’s identity? And if so would a facial mimetic-like response indicate such reinstatement—i.e., could rapid and sub-perceptual reactivation of the zygomaticus be detected? If this were to occur even if the individual being perceived was now not smiling themselves, this would support the notion of a reactivation of prior simulation.

The present study addresses this question. Participants were exposed to faces that consistently smiled or frowned, whilst the activity of their own facial muscles was recorded using facial EMG. Later, the participants were re-exposed to the same faces whilst completing a simple oddball task, and again, facial EMG was recorded. Importantly, however, at re-exposure, all the faces now had a neutral facial expression. Covert facial mimicry responses recorded during initial exposure to the emotional versions of the faces would indicate the presence of sensorimotor simulation. However, could reinstatement of these motor states be detected when the participants viewed the neutral versions of the face? If so this would suggest the reactivation of the prior simulation upon recalling the facial identity. A second issue was also addressed. Facial mimicry appears to occur even when the facial expression being viewed is not relevant to the task in hand. Similarly, reactivation of sensory or motor regions active during encoding of episodic memories occurs even when the sensory aspect of the memory is not being actively recalled (e.g., Vaidya et al. 2002). In the present study, the question of whether task relevance would affect the reactivation of facial mimicry was addressed by having half the participants attend to the emotional aspect of the faces they viewed, whilst the other half ignored the emotion and attended to the identity of the faces.

## Method

### Participants

Participants were 36 female undergraduates from Bangor University, recruited in two cohorts of 18 participants. The first cohort completed the attend to identity condition (mean age = 21.9 years, SD = 2.1 years), and the second cohort the attend to emotion condition (mean age = 19.9 years, SD = 2.7 years). All gave informed consent. Women appear more responsive than men in their physiological reactions to emotional facial expressions (Dimberg and Lundquist 1990). Therefore, a female cohort was selected as being more likely to demonstrate initial embodiment.

### Design

Participants completed three tasks. First, an initial learning task, during which face identities consistently expressed either smiling or frowning emotions and participants had to either identify oddball trials, where the identity of the face changed mid-trial, or identified the emotion expressed by the face. Here, it was predicted that participants would demonstrate facial mimicry, even though this was never an explicit task goal.

Second, a few minutes after the first task, participants completed an implicit recall task, where in the majority of trials, they passively viewed static, neutral versions of the faces seen during the learning phase. Their task was to monitor for occasional oddball targets, where either an identity change or an emotion change occurred, depending on the condition, they had been assigned to for the initial encoding task. It was predicted that during the passive viewing trials, implicit retrieval of the prior mimicry state associated with a face identity would occur if learning had taken place. Therefore, participants would show greater zygomaticus activity to those faces that had previously smiled that to those who previously frowned and greater corrugator activity to those faces that previously frowned than to those who previously smiled.

Finally, the participants completed an explicit recall task, viewing neutral versions of the previously seen faces, and making forced choice decisions as to whether the face had previously smiled or frowned.

In a mixed design, participants were divided into two groups. One attended to the emotion expressed on the faces during both the encoding, and implicit retrieval tasks. The other ignored the emotion and attended to the identity of the face.

### Stimuli

Morphing software was used to create dynamic facial expressions. Neutral, smiling, and frowning versions of four female and four male faces were selected from the Nim Stim (http://www.macbrain.org/mission.htm) and KDEF (Lundqvist et al. 1998) face sets. The eight identities used were selected to create four sex and attractiveness matched pairs, based on previously collected ratings of a larger set of faces, rated for attractiveness from 1 to 5. The male pairs were rated as 1.9 and 2.1, and 2.3 and 2.6. The female pairs as 2.7 and 2.9, and 3.7 and 3.9.

Dynamic morphs of each neutral face identity morphing into both expressions of every other face identity were also created, within sex. In total, this created 24 identity change morphs which were used as oddball trials in the initial learning task for participants tasked with attending to identity. For each participant, eight morphs were randomly selected, with each face appearing once as the end face of an oddball morph. A further set of two neutral male and two neutral female faces were also selected as oddball trials for use in the subsequent implicit retrieval task for the participants who were tasked with attending to identity.

### Procedure

After electrode placement, the participants completed the three tasks in the order described above. The participants were told that the study was about sustained attention and a story of frontal lobe recording was used to disguise the EMG measures. The participants were not told that the same faces would be seen throughout the experiment, or that any recall would be required.

#### Initial learning/encoding task (mimicry)

**Fig. 1:**
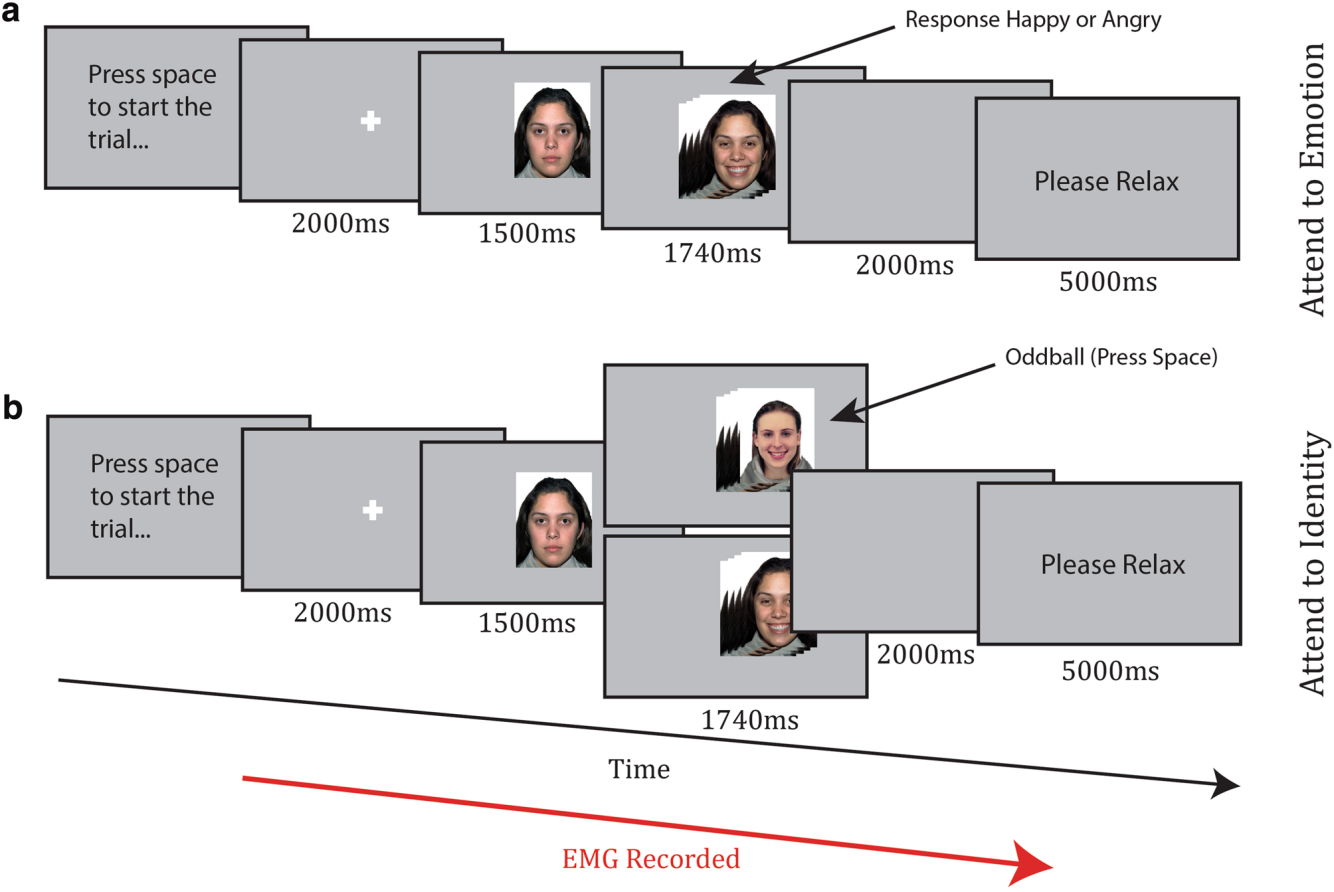
Timecourse of trials from **a** attend to emotion condition and **b** attend to identity condition of the encoding task

The procedure for the mimicry task can be seen in Fig. 1. Participants pressed the spacebar to begin each trial. A fixation cross appeared for 2000 ms. A face appeared, with a neutral expression lasting for 1500 ms, before morphing to an emotional expression (the transition took 240 ms), displaying a smile or a frown for 1500 ms. After a blank screen of 2000 ms, the participant relaxed for 5000 ms. All participants saw four identities (two males and two females) who always smiled, and four identities (two males and two females) who always frowned. Participants in the ‘attend to emotion’ condition (Fig. 1a, top panel) categorized the emotion that they saw in each face display during the trial, pressing either the Z or M key (counterbalanced). Participants in the ‘attend to identity’ condition (Fig. 1b, bottom panel) looked out for oddball trials, where a face would appear, then morph into the emotional face of another identity. Participants pressed the spacebar for oddballs, but did not respond to the standard trials. Oddball trials always involved faces morphing into another face of the same emotion category. Face allocation to emotion was quasi-random for each participant, so that there were always two smiling females and two smiling males and similarly two frowning males and females and that the attractiveness balance was maintained. The task consisted of 64 standard trials, divided into four blocks of 16. In the attend to identity condition, each block also contained two oddball trials, meaning a total of 72 trials.

#### Implicit recall task (neutral faces)

**Fig. 2:**
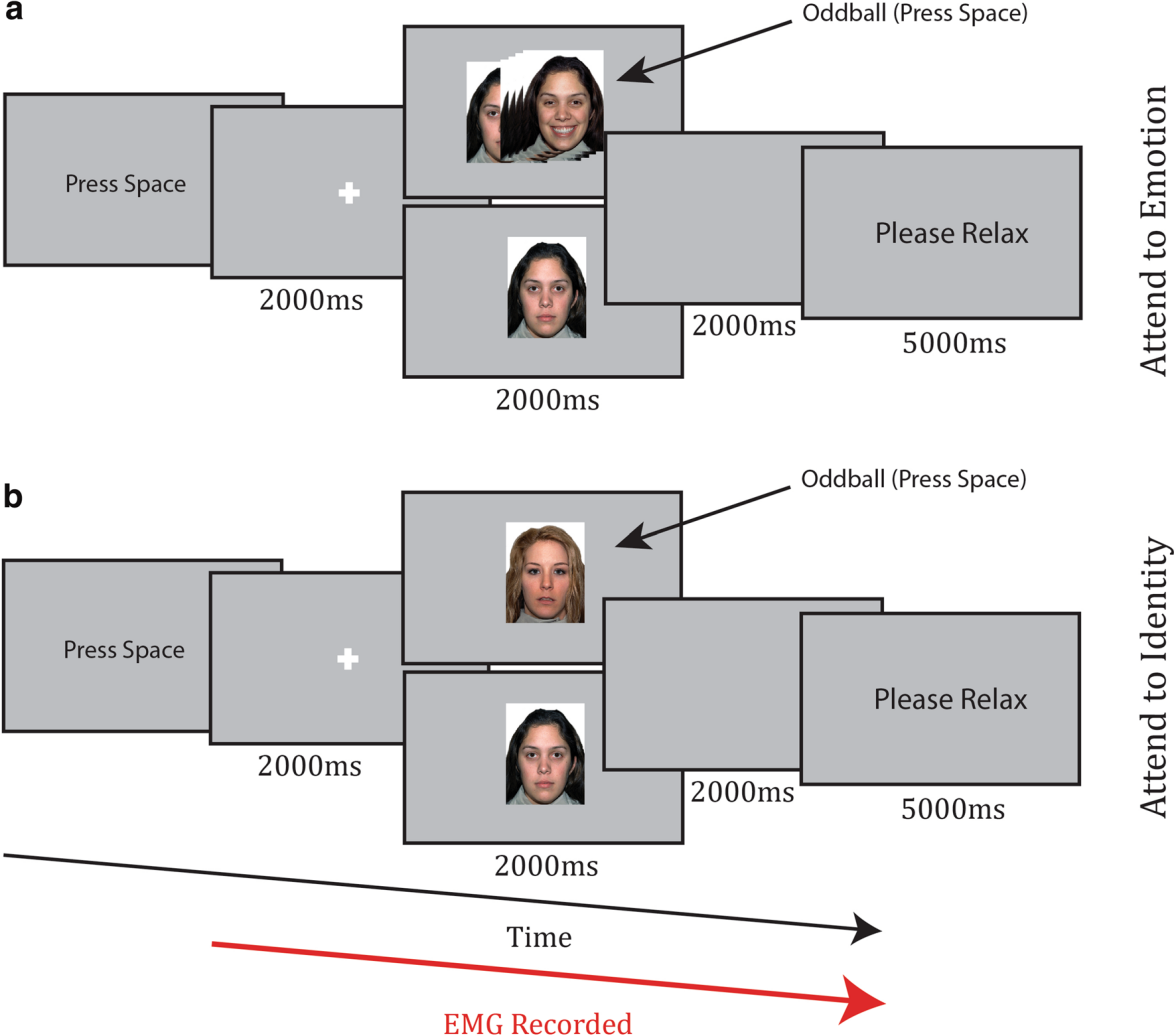
Timecourse of trials from **a** attend to emotion condition and **b** attend to identity condition of the implicit recall task

The procedure for the implicit recall task can be seen in Fig. 2. Participants pressed the spacebar to initiate trials, after which they were viewed a fixation cross for 2000 ms. A face appeared, with a neutral expression for 2000 ms. After this, the screen went blank for 2000 ms and the participant was asked to relax for 5000 ms. The task consisted of two blocks of 16 trials and each face identity appeared twice in each block. Each block contained two oddball trials. Oddballs in the attend to emotion condition (Fig. 2a, top) consisted of a face appearing for 1500 ms, then morphing to a smile or frown; there were always two smiling and two frowning faces, selected from those used during encoding and balanced for sex. In the attend to identity condition (Fig. 2b, bottom), oddballs were brand new identities with neutral expressions. In both conditions, participants responded to oddballs with a spacebar press and pressed nothing to the standard trials.

The selection of trial n’s in both tasks was guided by the number of trials used in the previous studies of facial mimicry (Dimberg and Thunberg 1998; Hess and Philippot 1998; Vrana and Gross 2004). We used a larger number of trials during the initial encoding task than during implicit recall to try and ensure the participants learned the emotions associated with each face.

#### Explicit recall task

Finally, participants were shown the eight faces seen in the previous tasks and made a forced choice decision (‘A’ and ‘L’ keys assigned to condition, counterbalanced) as to whether the face had smiled or frowned during the encoding task. Faces were presented once, with neutral expressions, for 2000 ms, and separated by a 2000 ms fixation cross prior to presentation and blank screen afterwards.

### EMG recording

EMG data were collected from the zygomaticus (smile muscle) and corrugator (frown muscle), using a BioPac MP100 system (BioPac: Goleta, CA). Data were sampled at 2000 Hz. Online filters were set to a bandpass of 1–5000 Hz and notch of 50 Hz. Electrode placement and preparation were conducted as guided by Fridlund and Cacioppo (1986). Recordings were made from the left side of the face (see Dimberg and Petterson 2000; Zhou and Hu 2004).

Offline data were filtered with a 20–400 Hz bandpass (as recommended in van Boxtel 2001), rectified, log10 transformed to reduce extreme values, and z-transformed within participant and muscle site. Change scores were calculated trial-by-trial. For every trial, the mean of the final 500 ms of the fixation was used as a baseline and subtracted from each data point during the trial, transforming each point into a change from baseline score. The data were averaged over 200 ms bins, using the arithmetic mean. These bins were used as the level at which data were visualized. For data analysis, data were averaged using the arithmetic mean, over whole trial periods (e.g., the period during which an emotional face was on screen, or the blank period after this). Artifact trials were removed by eye before the data were matched to condition. Error and post-error trials were removed from the EMG analysis to account for error-related activity (Elkins-Brown et al. 2016; Lindström et al. 2013) or post-error increases in effort (Van Boxtel and Jessurun 1993). The first trial of each task was removed upon observation of noisier data in these trials. EMG data from oddball trials were not analysed. For the encoding task, the mean number of (non-oddball) trials removed per participant was: attend to identity, happy faces 4.0 trials (SD = 1.5), and angry faces 4.7 trials (SD = 1.7); attend to emotion, happy faces 7.1 trials (SD = 2.2), and angry faces 5.6 trials (SD = 1.4). For the implicit recall task, the mean number of (non-oddball) trials removed per participant was: attend to identity, previously happy faces 1.3 trials (SD = 1.0), and previously angry faces 1.0 trials (SD = 1.1); attend to emotion, previously happy faces 2.1 trials (SD = 1.4), and previously angry faces 1.9 trials (SD = 1.1).

## Results and discussion

### Behavioral results for learning/encoding and implicit recall tasks

Accuracy rates were assessed for the encoding and implicit recall tasks. During encoding, accuracy in the attend to identity condition referred to the percentage of trials correctly categorized as oddballs if they were such, or not responded to if they were the standard trials. In the attend to emotion condition, accuracy referred to the percentage of trials correctly identified as containing a happy or angry facial expressions. In the implicit recall tasks, accuracy referred to the percentage of trials correctly identified as oddballs if they were such (new faces in attend to identity/emotional faces in attend to emotion), or not responded to if they were the standard trials. Accuracy rates were high across both the initial encoding and subsequent implicit recall tasks, suggesting that the participants were attentive to the stimuli. During the learning tasks, mean accuracies were above 98%, for both the attend to emotion and the attend to identity conditions. During the implicit recall tasks, accuracies were above 94% for both the attend to emotion and the attend to identity conditions.

### Mimicry during learning/encoding task

Facial mimicry was calculated using the EMG responses elicited by the emotion section of the morph videos, after the initial 1500 ms neutral expression, when the face became expressive, smiling, or frowning for 1500 ms. This period also included the 2000 ms blank screen after the offset of the face.

#### Corrugator (frown muscle)

**Fig. 3:**
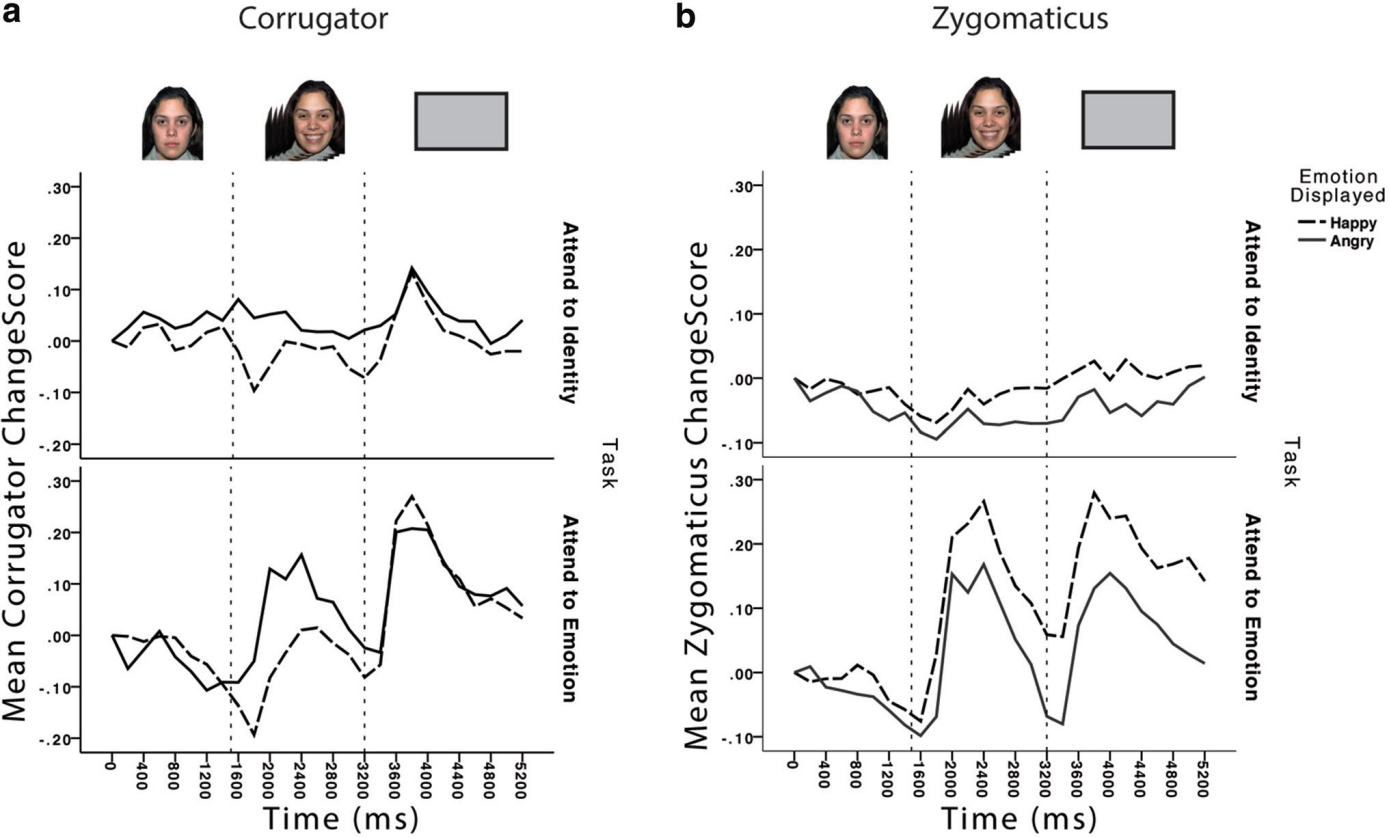
EMG responses during the encoding task from **a** corrugator and **b** zygomaticus muscles when the participants viewed faces smiling (happy) and frowning (angry). Changescores are standardised units of change from baseline resting state. *Dotted lines* represent transition points in the timecourse of the trial, as illustrated above the graph: after fixation, the face appeared with a neutral expression for 1500 ms, transitioned to an emotional expression which remained on screen for 1500 ms, before a blank screen appeared for 2000 ms

The activation states of the corrugator during and after the emotion expression face are shown in Fig. 3a. A three-way mixed analysis of variance was undertaken to analyse face emotion (happy/angry face), time (emotional face present/blank screen after face offset), and the between participants factor of task (attend to identity/attend to emotion).

Of primary interest are the significant results involving the face-emotion factor, which are indications of emotional mimicry. Of most importance, there was a significant main effect of face emotion, where as predicted, the corrugator was more active when viewing angry than when viewing happy faces, *F*(1,34) = 7.5, *p* = .01, $\eta _{p}^{2}$ = 0.180. Importantly, the mimicry of face emotion did not interact with whether participants were attending to the task relevant property of face emotion or irrelevant property of face identity, *F*(1,34) = 0.015, *p* = .902, $\eta _{p}^{2}$ < 0.001. Interestingly, there was also an interaction between face emotion and time, *F*(1,34) = 14.03, *p* = .001, $\eta _{p}^{2}$ = 0.292. Further analysis with uncorrected *t* tests revealed that the effect of face emotion was detected, whilst the emotional face was viewed, *t*(35) = 4.6, *p* < .001, but this effect was transient as there was no effect when the subsequent blank screen was viewed, *t*(35) = 0.73, *p* > .1.

The remaining significant effects did not involve the emotion factor, and thus indicate effects on the overall corrugator activation regardless of emotion. There was a significant effect of time, where corrugator activity increased from viewing faces to viewing the subsequent blank screen *F*(1,34) = 23.5, *p* < .001, $\eta _{p}^{2}$ = 0.408. The only significant between participants interaction effect was the interaction of task and time, *F*(1,34) = 7.8, *p* = .009, $\eta _{p}^{2}$ = 0.186. Follow-up uncorrected *t* tests revealed that whilst overall activity in the corrugator during the face and blank periods differed significantly for those participants who attended to emotion, *t*(17) = 5.1, *p* < .001, the difference was not significant for those who attended to identity, *t*(17) = 1.6, *p* > .1.

#### Zygomaticus (smile muscle)

The data are presented in Panel b of Fig. 3. The data were entered into the same structure of analyses as the corrugator data. Again, significant results involving the face-emotion factor are of primary interest. Of most importance, there was a significant main effect of face emotion *F*(1,34) = 7.05, *p* = .012, $\eta _{p}^{2}$ = 0.172, where the zygomaticus was more active when viewing smiling faces as compared to viewing angry faces, and this did not interact with whether attention was focused on face emotion or identity *F*(1,34) = 1.62, *p* = .289, $\eta _{p}^{2}$ = 0.033. Interestingly, there was no interaction in the zygomaticus between face emotion and time *F*(1,34) = 0.88, *p* > .1, $\eta _{p}^{2}$ = 0.055, meaning that, in contrast to the corrugator muscle, the mimicry effect remained stable into the blank screen period after the offset of the emotional face. Significant results not involving the face-emotion factor were a main effect of time *F*(1,34) = 7.09, *p* = .012, $\eta _{p}^{2}$ = 0.173, where activity increased across time from the period where the face was on screen into the blank screen after the offset of the face and a main effect of task *F*(1,34) = 12.4, *p* < .001, $\eta _{p}^{2}$ = 0.267, where the zygomaticus muscle was more active when attending to emotion than attending to identity.

Two key findings here are of note. First, even though participants were not asked to explicitly mimic the face emotion whilst attending to their particular task, the mimicry effect was still detected in both muscles. Second, this seems to be independent of the participant’s task, as there is no interaction between face emotion and task of attending to emotion or attending to face identity. That is, whether directly attending to and making decisions about face emotion, or ignoring the emotion whilst reporting occasional identity changes, the mimicry effect is observed. This supports the notion that mimicry is evoked without intent (e.g., Cannon et al. 2009; Dimberg et al. 2000, 2002).

It is also worth noting that across both muscle sites, the pattern of activity seen in the attend to emotion condition differed to that in the attend to identity condition. In the former, it appeared that mimicry constituted a greater increase in activity from baseline levels, whereas in the latter, it appeared as a decrease in activity from baseline to the incongruent expression. Finally, it worth noting that mimicry seems to be longer lasting in the zygomaticus muscle, continuing in to the blank field after face offset; whereas in contrast, mimicry is only detected in the corrugator muscle whilst participants are actually viewing an emotion expressing face.

The next analysis examined the EMG data from the implicit recall section of the experiment, where the participants viewed the faces that had previously smiled or frowned, but this time showed neutral expressions. Here, we predicted that there would be a reactivation of the facial mimicry responses detected in the encoding task.

### Implicit recall task (neutral faces)

A three-way mixed analysis of variance was carried out on these data, within each muscle site, examining face emotion (previously appeared happy/previously appeared angry), time (neutral face present/ blank screen), and task (attend to identity/attend to emotion).

#### Corrugator

**Fig. 4:**
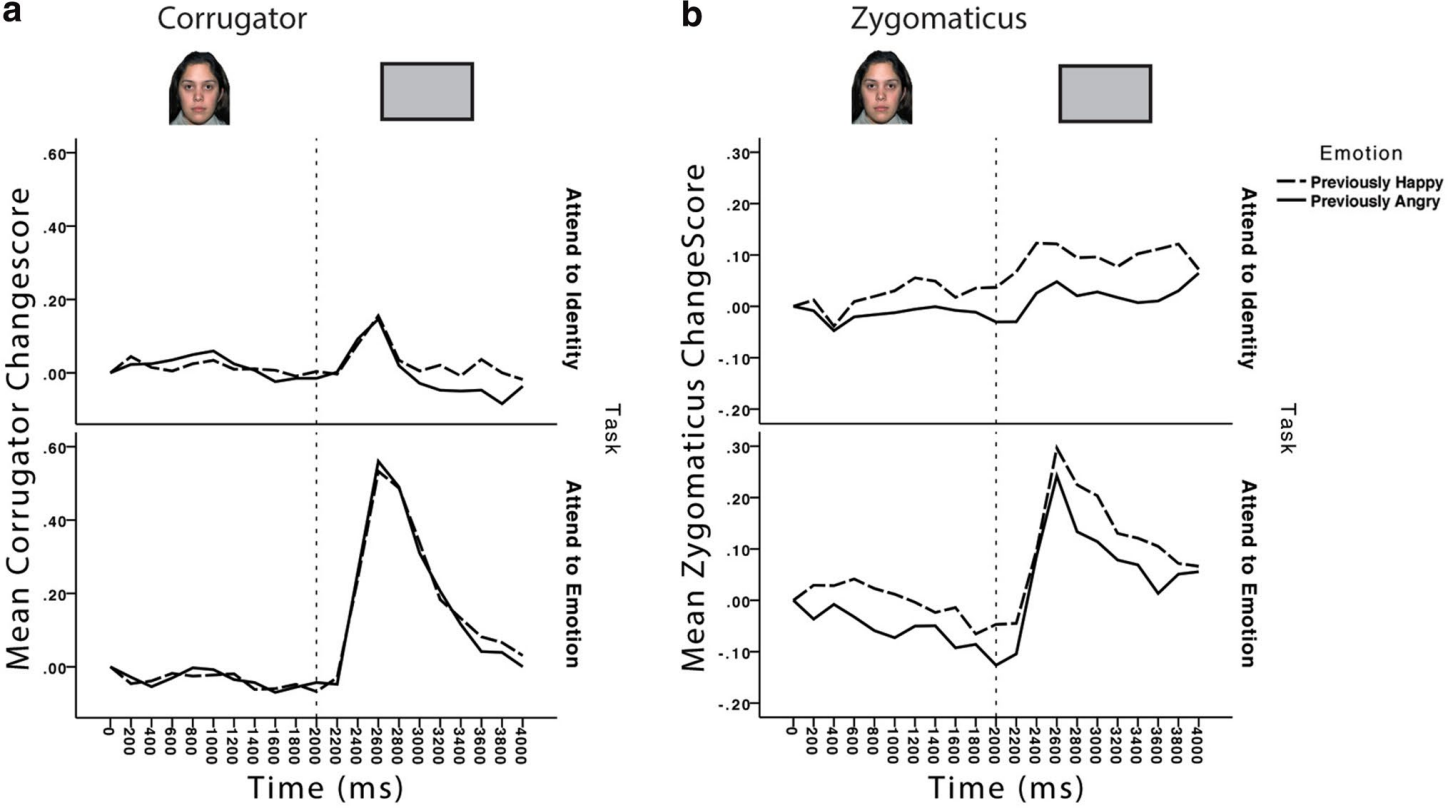
EMG responses during the implicit retrieval task from **a** corrugator and **b** zygomaticus muscles when participants viewed neutral faces. Changescores are standardised units of change from baseline resting state. *Dotted lines* represent transition points in the timecourse of the trial, as illustrated above the graph: after fixation, the face appeared with a neutral expression for 2000 ms, before a *blank screen* appeared for 2000 ms

The data are presented in Fig. 4a. Importantly, there was no main effect or interactions involving prior face emotion; this indicates that retrieval of prior emotional mimicry did not occur. The only significant main effects and interactions involved the factors of task and time. Participants showed more corrugator activity during the blank screen period than during the period, where the face was onscreen, *F*(1,34) = 18.7, *p* < .001, $\eta _{p}^{2}$ = 0.355, but this varied between those participants attending to emotion and those attending to identity, *F*(1,34) = 18.6, *p* < .001, $\eta _{p}^{2}$ = 0.353. Uncorrected *t* tests conducted to explore this interaction revealed that the increase in activity from face to blank period was only significant in those participants attending to emotion, *t*(17) = 4.6, *p* < .001. Overall, the participants attending to emotion showed greater corrugator activity as well, *F*(1,34) = 8.3, *p* = .007, $\eta _{p}^{2}$ = 0.195.

Therefore, the corrugator provides no evidence for retrieval of prior facial emotion states. This might not be entirely unexpected given that previous studies have also observed less robust effects in corrugator than in zygomaticus muscles (e.g., Cannon et al. 2010) and have reported less robust mimicry of the socially costly expression of anger, than on less costly expressions, such as happiness and sadness (Bourgeois and Hess 2008; Hess and Bourgeois 2006; but see; Kirkham et al. 2015, for contrasting effects).

#### Zygomaticus

The data are presented in Fig. 4b. Most importantly, there was a main effect of prior face emotion, *F*(1,34) = 7.7, *p* = .009, $\eta _{p}^{2}$ = 0.184, with increased zygomaticus activation when viewing a neutral face that had previously expressed a positive/smiling emotion as compared to a neutral face that had previously expressed anger and this did not interact with the face property attended (emotion or identity), *F*(1,34) < 0.1, *p* = .9, $\eta _{p}^{2}$ < 0.1. The remaining significant effects did not involve the face-emotion factor. There was a main effect of time, *F*(1,34) = 23.7, *p* < .001, $\eta _{p}^{2}$ = 0.410, with activation increasing from the face to blank periods. Although the interaction of task and time was significant, *F*(1,34) = 5.6, *p* = .02, $\eta _{p}^{2}$ = 0.142, follow-up uncorrected *t* tests revealed a significant increase in zygomaticus activity from the face to blank periods in both participants attending to identity, *t*(17) = 2.9, *p* = .01, and emotion, *t*(17) = 4.0, *p* = .001.

### Explicit recall task

At the end of the study, participants were presented with the faces and asked to recall whether the person had previously smiled or frowned. Data from two participants in the attend to identity condition were missing from this analysis due to experimenter error in saving the data and a participant misunderstanding the key presses. Mean percentage accuracy (correct identification of whether a face previously smiled or frowned) was high amongst both those participants attending to emotion (mean = 95.1%, SD = 7.6%) and those attending to identity (mean = 82.03%, SD = 20.4%). Both the attend to emotion (*p* < .001 for *t* test against 50%) and the attend to identity (*p* < .001) groups performed above chance on this task, but, perhaps predictably, a comparison of the two groups’ performance showed that the group who attended to emotion recalled significantly more faces’ prior emotions correctly, *F*(1,32) = 6.4, *p* = .016. It is also worth noting that the attend to identity group also saw oddball trials at encoding, which might have interfered with learning.

### Individual differences in mimicry

Finally, we make a tentative analysis concerning individual differences in mimicry processes. Although mimicry of another person’s emotion is a robust effect observed in a number of previous studies, not all people produce detectable mimicry (see Manssuer et al. 2016, for other evidence for individual differences in evoked emotion and learning). This could have implications for later retrieval of prior body states, in that people who did not initially mimic whilst observing faces expressing emotion cannot subsequently retrieve and reestablish a mimicry state.

Therefore, we divided participants into those who mimicked observed emotion in the first stage of the study and those who did not mimic. This was done by separating those participants who showed muscle activity trending in the expected direction in each muscle from those who did not. Therefore, for analysis of the zygomaticus, we compared those participants who showed greater activity to smiles than frowns (mimickers) to those who showed the opposite (non-mimickers). In addition, in the corrugator, we did the opposite, comparing those who showed greater activity in response to frowns (mimickers) to those who showed greater activity in response to smiles (non-mimickers). This does not mean that the participants in the mimicry group mimicked every face, and only that on average, they showed an effect that suggested mimicry. We predicted that retrieval of mimicry in the later retrieval stage can only be detected in the individuals who originally mimicked, and whilst no significant effects were found in the corrugator analysis, this was indeed the case for the zygomaticus muscle [*N* = 25; *F*(1,24) = 9.7, *p* = .005, $\eta _{p}^{2}$ = 0.287]. In sharp contrast, those who did not mimic the observed emotion in the initial stage of the study also did not produce any evidence of mimicry in the later retrieval stage [*N* = 11, *F*(1,10) = 0.011, *p* = .92, $\eta _{p}^{2}$ = 0.001]. On the other hand, when examining explicit recall of prior emotion, the mimickers (mean accuracy = 90.2%, test against chance *p* < .001) and non-mimickers (mean accuracy 86.4%, test against chance *p* < .001) performed similarly. Hence, explicit recall is not reliant on implicit retrieval of prior body states (e.g., Topolinski 2011). Clearly, the small sample size in the latter non-mimicry group requires that we are cautious and tentative with our conclusions, but retrieval of prior embodied states predicts just such a result, and hence, it is worthy of future investigation.

## General discussion

The current study provided a number of new observations that add to the discussion surrounding the role of sensorimotor simulation in social cognition (Wood et al. 2016). First, automatic mimicry of another person’s emotion was again observed in this study. Thus, even though participants were not asked to overtly mimic another person’s emotion, mimicry was nevertheless detected through the use of facial EMG. This replicates previous research (e.g., Cannon et al. 2009; Dimberg et al. 2000, 2002), and fits theory purporting that facial motor regions are vicariously activated when viewing facial expressions (Wood et al. 2016).

Furthermore, the mimicry effect was observed both when emotion was relevant and irrelevant to the participant’s goal, supporting the notion that facial mimicry is automatic, and can to be evoked even when another person’s emotion is task irrelevant and not explicitly attended to (e.g., Cannon et al. 2009; Dimberg et al. 2000). It is worth noting that the patterns of muscle activity differed between the attention conditions. When attending to emotion, facial mimicry in both muscles appeared as a larger increase from baseline activity. However, in the attend to identity condition, it took the form of a lesser reduction from baseline activity; for example, the zygomaticus activity decreased from baseline to a lesser degree for happy than angry faces. Similar results have been reported in other experiments (see Cannon et al. 2009; Dimberg et al. 2000). It might be that the pattern of activity in the attend to identity condition is indicative of a greater preparatory response in both muscles, prior to the presentation of the face—perhaps because the task required more effort. Or, as suggested in Cannon et al. (2009), it could be due a suppression of the task irrelevant emotion.

However, the core issue engaged here was the reactivation of simulation during later encounters. Embodied accounts of emotional memory processes (e.g., Niedenthal 2007) propose that perceptual-motor states are encoded during initial experience of a stimulus. In the current case, this would be the activation of facial muscles whilst viewing another person express an emotion, which in accordance with simulation models, results from vicarious neural activity in facial motor regions. We predicted that such body states might be reactivated when encountering a person again, even if they were not expressing any emotion. We found that indeed, there is evidence for such reinstatement of prior processing, which we saw in the zygomaticus muscle. Here, muscle activity reflecting mimicry during previous encoding of emotional faces can be detected when the faces are later viewed with neutral expressions. Interpreted in the light of current theory this supports the notion that sensorimotor simulations occurring during encoding are reinstated during recall. This finding aligns with models of emotional memory and with evidence that during episodic memory retrieval, states of sensory and motor neural activity that were active at encoding are reactivated. Our data fit particularly well with evidence that neural simulations of actions encoded alongside verbal stimuli are reactivated upon later retrieval of the verbal information alone (Nyberg et al. 2001).

However, there appear to be boundary conditions. First, the retrieval effects were only detected in the zygomaticus muscle and not the corrugator, despite initial mimicry in both muscles. It may be the case that the more subtle embodied responses are simply detected more easily in the zygomaticus (see Cannon et al. 2010). It is also perhaps noteworthy that the mimicry activity in the corrugator in the present study appeared fleeting, only differentiating between angry and happy faces when the faces were onscreen. Prior research has indicated that facial mimicry is influenced by social context (for review, see Hess and Fischer 2014), particularly the more socially costly mimicry of anger. It might be the case that reactivation of the corrugator inhibited by either potential social cost or because of reduced affiliation with faces who previously demonstrated consistently negative expressions (Bourgeois and Hess 2008).

In an exploratory analysis, we compared reactivation effects in the zygomaticus between participants who did and did not demonstrate mimicry in this muscle. Interestingly, whilst mimickers and non-mimickers showed an equal ability to explicitly recall the face-emotion pairings, it was only amongst the mimickers that reactivation of mimicry was seen. The scope for conclusions on this result is limited by the small sample size and simplistic division of mimickers and non-mimickers. However, the results are none-the-less interesting and suggest that further exploration of individual differences in the utilization, or outward expression of reactivated simulations is needed. It is also worth noting the contrasting results of a similar study (Kirkham et al. 2015). Here, during encoding, participants viewed emotional faces who smiled and frowned in a manner that was either consistent or inconsistent with an emotional context, created by the simultaneous presentation of an emotional image alongside the face. Mimicry was reduced for faces whose expressions were inconsistent with the emotional context. However, in a later retrieval phase where the faces still smiled and frowned, but no emotional context was presented, the prior consistency or inconsistency of a face’s expressions did not affect participant’s mimicry—i.e., no retrieval of prior emotion consistency was found. Here, unlike the current study, which used neutral faces at retrieval, the facial mimicry evoked at retrieval may have blocked any detection of prior mimicry states being reactivated.

Finally, some limitations of the current study and possible future directions require discussion. Numerous experiments have explored the role of sensorimotor simulations and facial mimicry in accurately decoding facial expressions (see Ipser and Cook 2016). The current study did not utilize a design that allowed for such an analysis of the role that reactivation of simulations might play. For example, simulations might be retrieved as part of predicting the other person’s oncoming emotion (Heerey and Crossley 2013), or might play a part in impression formation (Halberstadt et al. 2009). We did not record EMG activity during explicit recall, where reactivation of facial mimicry might have predicted accurate retrieval of prior emotion condition. We also used a relatively small number of faces, and an extension using a larger face set might be better suited for exploring links between reactivated facial mimicry and impression formation or accurate emotion recall.

It would also be interesting to investigate similar effects with non-facial stimuli that elicit mimicry-like responses, such as emotional bodies (Tamietto et al. 2009), or images (Dimberg 1986). It is difficult in the present study, but also in the literature as a whole, to decipher whether facial mimicry responses constitute simulation, emotion contagion, or just emotional response (Hess and Fischer 2014). Perception deficits specific to perceiving facial emotion when blocking facial mimicry, for example, support the notion of mimicry as simulation (Ipser and Cook 2016; Neal and Chartrand 2011). However, it would be enlightening to explore similar boundaries on the retrieval of mimicry. For example, would blocking later reactivation of mimicry cause a decreased accuracy in remembering prior emotions?

We sampled only female participants, and therefore, our results can only be generalized to the female population. Examining male participants might help cast light on the individual differences that might underlie the apparent link seen in our exploratory analysis between initial mimicry and later mimicry reactivation. We also used faces that always smiled or frowned. Of course in the real world, people hardly ever express a single emotion consistently, and hence, a more ecologically valid follow-up could include identities that emote less predictably.

To conclude, we have confirmed that mimicry of another person’s emotion takes place without intention and even when attending to irrelevant properties of a face, such as identity. Of most importance, in support of simulation accounts of memory, we show encoding of the relationship between a person’s identity and their typical emotion, via reactivation of prior motor simulation. That is, mimicry evoked whilst viewing a person expressing emotion can be reactivated at a later time, even when they are no longer expressing any emotion. In addition, although tentative at this time, it appears that such reactivations only occur in individuals who demonstrate mimicry responses in the first instance.
